# Wastewater Surveillance of Mpox during the Summer Season of 2023 in Slovenia

**DOI:** 10.3390/idr16050065

**Published:** 2024-08-29

**Authors:** Jan Rožanec, Natalija Kranjec, Ivana Obid, Andrej Steyer, Tjaša Cerar Kišek, Tom Koritnik, Mario Fafangel, An Galičič

**Affiliations:** 1 National Institute of Public Health, Trubarjeva ulica 2, SI-1000 Ljubljana, Slovenia; 2 National Laboratory of Health, Environment and Food, Prvomajska ulica 1, SI-2000 Maribor, Slovenia

**Keywords:** mpox, wastewater-based epidemiology, emerging pathogens, PCR analysis, public health interventions

## Abstract

Since COVID-19, mpox was the first emerging pathogen to have spread globally in 2022. Wastewater-based surveillance (WBS) has proven to be an efficient early warning system for detecting potential resurgences. This report aims to provide insight into the development and implementation of WBS of mpox in Slovenia and to incorporate the surveillance results into the development of public health interventions. WBS of mpox was conducted during the period from 1 June 2023 to 30 September 2023 at the wastewater treatment plant (WWTP) Ljubljana and WWTP Koper. The selected detection method of the monkeypox virus (MPXV) in the wastewater sample was based on PCR analysis. The implemented laboratory method showed that the sample preparation and concentration method enables a stable procedure for MPXV detection in wastewater samples. The laboratory analysis of wastewater samples from the selected WWTPs did not detect the MPXV during the monitoring period. In the event of MPXV detection in a wastewater sample, targeted public health interventions would be implemented, focusing on increasing awareness among the groups of men who have sex with other men and searching for positive mpox cases. We recommend that the developed system be retained in the case of an emergency epidemiological situation.

## 1. Introduction

The 2022 mpox outbreak, which was reported from countries globally, had 99,176 confirmed cases of mpox in 116 countries by 30 June 2024, of which 27,529 cases were reported in the World Health Organization (WHO) European Region [1], with 47 of those in Slovenia [2]. In Slovenia, we have reported positive clinical mpox cases in the period from 18 May 2022 to 20 September 2022, after which there have been no new cases of mpox detected with clinical surveillance up to the end of July 2024. The affected population in Slovenia consisted exclusively of men aged 20 to 61 years (mean age 38 years), where 37 cases (79%) were likely to have been caused by the sexual transmission of mpox [2]. According to the WHO [3] and other research studies [4,5,6,7], the majority of infected people with mpox are men who have sex with other men (MSM).

Mpox viral DNA can be detected in urine, stool, saliva, skin lesions, and mucosal lesions as well as in semen samples of confirmed mpox cases [5,8,9], with 67% of infected people shedding the virus via stool and 31% via urine [5]. Further, the monkeypox virus (MPXV) can be shed by an infected person, regardless of whether they are symptomatic or asymptomatic [8,10]. These findings indicate that wastewater surveillance (WWS) of mpox is technically feasible. Therefore, WWS can be used to identify the potential circulation of the MPXV in the population and can also provide a rough estimate of the trend of mpox prevalence in the monitored population by analysing mpox viral DNA concentrations [11,12]. Wannigama et al. [10] found that WWS of mpox can detect the circulation of MPXV in a population before the first confirmed clinical mpox case.

WWS of mpox has already been implemented in the first year of the outbreak, and the presence of the MPXV in wastewater has been detected in several countries [10,11,12,13,14,15,16,17,18,19]. Due to the global mpox outbreak in 2022, the WHO [9] also encouraged countries to support research on WWS for mpox to clarify possible objectives, approaches, methods, and challenges for this type of surveillance.

In spring 2023, the mpox epidemiological situation for the upcoming summer season was unpredictable, and mpox cases continued to occur in Europe and globally [20], while social life returned to pre-pandemic levels after three years of restrictions due to the COVID-19 pandemic. In the second half of 2022, mpox vaccination was already introduced for the people of the susceptible population group [21], thereby establishing a certain level of immunity among the susceptible population. In Slovenia, a total of 550 people were vaccinated against mpox by 31 May 2023 [22]. In a susceptible population, there was also established high awareness of mpox, due to the knowledge of the disease, which might otherwise lead to underreporting of clinically confirmed mpox cases, or to seeking medical care only in advanced stages of mpox disease, due to the stigma associated with sexually transmitted diseases [10,15,23]. Many people develop only mild symptoms, which can also contribute to possible underdiagnosis and result in an underestimation of the actual number of clinically confirmed mpox cases [6,8,24]. For these reasons, which suggested the unpredictable spread of mpox during the summer season, WWS for mpox was also implemented in Slovenia. Therefore, this study aims to provide insight into the rapid development and implementation of wastewater-based epidemiological surveillance of mpox in Slovenia and to incorporate the surveillance results into the development of public health interventions. We have set the following objectives: (a) development of a laboratory method for the detection of the MPXV in wastewater samples, (b) monitoring the epidemiological situation in Slovenia regarding the MPXV in the summer season of 2023, and (c) implementation of WWS for mpox as an important source for the development of public health interventions.

## 2. Materials and Methods

### 2.1. Mpox Monitoring Plan

The WWS of mpox in Slovenia was carried out during the summer season of 2023, from 1 June 2023 to 30 September 2023. The selection of the monitoring period was based on three data-based scientific considerations. Firstly, during the summer season of 2022, the highest number of mpox cases were reported globally, with a global peak of 31,075 monthly cases observed in August 2022 [3]. Secondly, during the monitoring period, the highest number of mass gatherings was organised in Slovenia [25], which could have a potential impact on the occurrence and further spread of the MPXV among the population. Finally, during the monitoring period, the peak summer tourist season in Slovenia took place [26].

**Figure 1:**
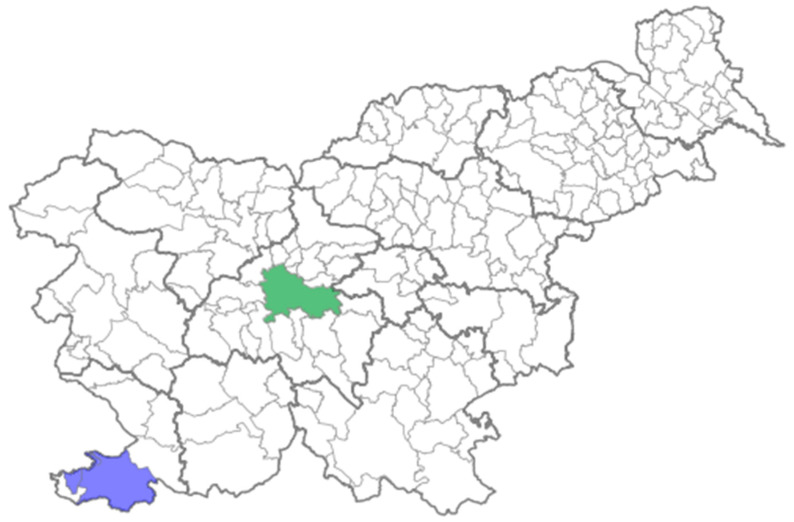
Catchment areas of the wastewater treatment plant Ljubljana (green colour) and the wastewater treatment plant Koper (blue colour), included in the wastewater surveillance of mpox in Slovenia, during the summer season of 2023.

WWS of mpox was implemented at two wastewater treatment plants (WWTPs); the WWTP Ljubljana, which covers the municipality of Ljubljana, and the WWTP Koper, which covers the municipalities of Koper, Izola, and Ankaran. The municipality of Ljubljana has the largest population of all municipalities in Slovenia (297,575 inhabitants), which includes Ljubljana, the capital city of Slovenia. While Koper is the fourth largest municipality in Slovenia in terms of population (54,252 inhabitants), Izola and Ankaran are smaller municipalities (16,479 and 3325 inhabitants) [27]. The catchment area covered by the included WWTPs in the WWS of mpox is presented in Figure 1.

The wastewater monitoring locations were selected based on the identified high-risk areas for mpox infections, taking into account the locations of the existing WWS system. The selection of the wastewater monitoring locations was based on five data-based scientific considerations. Firstly, in Ljubljana, 49% of all cases in Slovenia were detected in 2022, which represents the highest proportion of confirmed mpox cases among all Slovenian municipalities [2]. Secondly, Ljubljana hosts several events attended mostly by members of MSM groups. Thirdly, within the catchment area of the WWTP Ljubljana and WWTP Koper, there are many known locations where members of MSM groups gather. Fourthly, Ljubljana was the Slovenian municipality with the highest number of tourist arrivals (490,516 tourist arrivals) and the second-highest number of overnight stays among Slovenian municipalities (1,128,627 overnight stays) in the period from June to September 2022. Likewise, the municipalities of Koper, Izola, and Ankaran had a high number of tourist arrivals and overnight stays, with 195,120 tourist arrivals and 814,630 overnight stays in the observed period [26]. Finally, the catchment population of the WWTP Ljubljana and WWTP Koper covers a large proportion of the municipalities’ population: above 70% in each of the municipalities of Ljubljana, Koper, Izola, and Ankaran. As required by national legislation, all municipalities have accommodation, and public buildings connected to the public sewerage system.

### 2.2. Sampling and Transport of Samples

The sampling was performed using a 24 h composite sampling with an automatic flow-proportional sampler. After pre-setting the automatic sampler, a representative sampling of the influent wastewater was carried out over a period of 24 h, taking partial samples in a total volume of 1 L. The sample was transported to the laboratory in sterile packaging at a temperature of 4 to 8 °C and stored at this temperature until the start of analysis. The analyses in the laboratory started within 48 h of the sample collection.

### 2.3. Implementation of the Microbiological Diagnostics

To assess the analytical performance of the detection method, we conducted a spiking experiment using the inactivated MPXV of clade I, generously provided by the Friedrich-Loeffler-Institut. Prior to spiking, 1 L of influent 24 h composite sewage samples, previously confirmed negative for MPXV, was divided into twelve aliquots: six samples of 100 mL (each comprising 2 × 50 mL) and six samples of 35 mL each. Additionally, to serve as process control, all wastewater samples were spiked with the Equine Arteritis Virus (EAV). Nucleic acid extraction was performed from the collected solid and liquid phases of the samples.

Tenfold dilutions of the MPXV were prepared and spiked into sewage samples to achieve tenfold dilutions from 1:500 to 1:5,000,000. Each spiked sample was thoroughly mixed. Care was taken to prevent cross-contamination between samples during handling and preparation.

Spiked samples of 100 mL were centrifuged for 30 min at 3200× *g* at 4 °C. The resulting sediments were used for nucleic acid isolation. Total nucleic acids were extracted using an automated procedure on the QiaCube instrument, using the AllPrep PowerViral DNA/RNA Kit (cat. No 28000-50, Qiagen, Hilden, Germany). To improve lysis, a bead-beating option with 0.1 mm Glass PowerBead Tubes (cat. No 13113-50, Qiagen, Hilden, Germany) was used.

Spiked wastewater samples of 35 mL were processed using an affinity-based capture method with magnetic hydrogel Nanotrap particles with Enhancement Reagent 1 (cat. No 10111-10, Ceres Nanosciences, Manassas, VA, USA). First, 100 μL of Ceres Nanotrap Enhancement Reagent 1 (Ceres Nanosciences Inc., Manassas, VA, USA) was added to each sample and inverted two times to mix. Subsequently, 525 μL of Nanotrap Microbiome A Particles (Ceres Nanosciences Inc., Manassas, VA, USA) was added and mixed via two inversions. Samples were incubated on a rotator at room temperature for 30 min. Samples were placed on the DynaMag^™^-50 Magnet (cat. No 12321D, Thermo Fisher Scientific, Waltham, MA, USA) for 10 min to separate the Nanotrap particles from the wastewater. The supernatant was carefully removed with a pipette without disturbing the particle pellet. A total of 1 mL of molecular biology-grade water was added to the tube and the particle pellet was resuspended by pipetting. The suspension was transferred to a 1.5 mL microcentrifuge tube and placed on a DynaMag-2 magnetic rack for 2 min. After removing the supernatant, 400 μL of MagMAX Wastewater Ultra Nucleic Acid Isolation Kit (cat. No A52610, Applied Biosystems, Waltham, MA, USA) Lysis Buffer was used for re-suspending the particle pellet. After 10 min of incubation at room temperature, the microcentrifuge tube was placed on a DynaMag^™^-2 magnetic rack for 2 min. Then, 400 μL of supernatant was transferred to a new 2 mL collection tube.

Nucleic acids were extracted from each concentrated sample using the MagMAX Wastewater Ultra Nucleic Acid Isolation Kit (cat. No A52610, Thermo Fisher Scientific Inc., Waltham, MA, USA) on the KingFisher Flex automatic extraction system (cat. No 5400630, Thermo Fisher Scientific Inc., Waltham, MA, USA). For every extraction, a negative control (PCR-grade water) was used.

A LightMix^®^ Modular Monkeypox Virus kit (cat. No 58-0550-96, Roche, Mannheim, Germany) was used for specific detection of the MPXV. The assay targeted the J2L/J2R and B7R gene regions of the MPXV, with a manufacturer-reported limit of detection (LOD) of <10 copies/rxn.

For detection of the EAV, a LightMix^®^ Modular EAV RNA Extraction Control kit (cat. No 66-0909-96, Roche, Mannheim, Germany) was used. Undiluted and 1:10 diluted extracted nucleic acid samples were tested in duplicates. The PCR reaction mix was prepared using 9.5 µL PCR-grade water, 5 µL TaqMan Fast Virus 1-Step Master Mix (cat. No 4444436, Thermo Fisher Scientific Inc., Waltham, MA, USA), and 0.5 µL of primers and probe mix. Synthetic cloned fragments included in the kit served as positive controls.

We applied the same procedures to analyse wastewater samples collected from the field. Briefly, all wastewater samples were aliquoted in 100 mL and 35 mL portions, and the EAV was added as a process control. Samples of 100 mL were centrifugated, and nucleic acid was extracted from solids; samples of 35 mL were subjected to affinity-based capture using Nanotrap particles. qPCR detection of the MPXV and the EAV was carried out using the same LightMix^®^ Modular kits (cat. No 58-0550-96 and cat. NO 66-0909-96, Roche, Mannheim, Germany) and PCR reaction setup as described earlier. Undiluted and 1:10 diluted samples were tested in duplicates. Negative controls were included in each extraction, and qPCR was run to monitor for potential contamination. Samples were considered positive if their Ct values were below 37. In accordance with PCR methodology, higher Ct values appear in samples with lower concentrations of the target abundance and vice versa.

## 3. Results

Using a spiking experiment, we demonstrated that our sample preparation and concentration method provides a stable procedure for the detection of the MPXV in wastewater samples (Table 1). In spiked and concentrated total wastewater samples, the Ct values ranged between 20.5 and 30.1 for mpox inoculum dilutions from 1:500 to 1:500,000. Our experiment showed that the MPXV concentration was higher in the 35 mL total wastewater samples compared to the sediment fraction only, obtained from a 100 mL wastewater sample. The Ct difference was between 5.81 and 9.38. The process control was detected with comparable Ct values in all samples and was also detected in lower concentrations (higher Ct values with Ct differences between 6.09 and 7.95) in sediment fractions. Although the method is not intended to be quantitative, it serves as a reliable detection method. It is important to note that a negative result does not guarantee the absence of the MPXV in the analysed sample.

The laboratory analysis of wastewater samples from the epidemiological surveillance of mpox from the WWTP Ljubljana and WWTP Koper did not show positive results for MPXV during the monitoring period. The results of PCR analysis of wastewater samples from the epidemiological surveillance of mpox in Slovenia for the monitoring period are shown in Table 2.

## 4. Discussion

The WWS of mpox carried out from 1 June 2023 to 30 September 2023 in Slovenia did not detect any positive wastewater samples for MPXV, which is consistent with the results from surveillance of the clinical data, with the last positive case of mpox detected in 2022 [2]. Meanwhile, clinically confirmed cases of mpox from the year 2023 until 30 September 2023 were reported from three neighbouring countries: Italy (25 clinically confirmed cases), Croatia (4 clinically confirmed cases), and Austria (3 clinically confirmed cases) [3]. The WWS of mpox in Slovenia has only been conducted at two WWTPs, which could potentially pose a risk of undetected sporadic cases of mpox in areas where no wastewater monitoring has been carried out. However, even though WWS was only conducted at two WWTPs, the areas with the highest identified risk for mpox infections in Slovenia were included in the surveillance.

When no virus is detected in wastewater samples, it means that the population in the monitored area is either not shedding viral particles or that the amount of viral DNA in the sample is below the detection limit [13]. During the implementation period, several possible primer pairs were investigated. The selected kit from TIB Molbiol (LightMix Modular Monkeypox Virus) has been shown to have a low LOD, no cross-reactivity, and comparable Cq values for the tested samples with other pox viruses, making it a suitable choice [28]. One of the possible disadvantages of this study is the complex nature of the samples. Many wastewater samples have a lot of PCR inhibitors [29]; however, process and internal controls were used, and 1:10 dilutions of all samples were tested in parallel with the undiluted samples to minimise the possible effects of any PCR inhibition occurring.

In this study, we performed nucleic acid extraction from the collected solids and the concentrated total wastewater sample. Previous studies demonstrated that the concentration of mpox is significantly higher in the solid phase of wastewater [11]; however, the liquid phase cannot be disregarded as many authors also detected the MPXV from liquids [14,15]. The sensitivity of the detection assays varies with the amount of people shedding MPXV. For example, Adams et. al. [30] found that the sensitivity of the assays ranged from 14% to 77% depending on the number of persons shedding MPXV. Further, the vaccination among a susceptible population in Slovenia could have affected the shedding of MPXV in infected individuals; however, the impact cannot be estimated because we do not know the proportion of the vaccinated susceptible population, 94% of individuals were vaccinated due to an epidemiological indication [22], and current data on the effect of vaccination and drugs on the shedding in infected individuals are limited [31]. Another disadvantage of the approach taken in this monitoring campaign is the frequency of the sampling, as sampling several times per week could increase the sensitivity of detection in a low-prevalence area. Adams et. al. [30] found that sensitivity increased with increasing sampling frequency by 14% for the daily sample and 32% for weekly sensitivity (several daily samples in a week). Furthermore, the number of people shedding MPXV also affects the probability of detection. When one person was shedding MPXV, Adams et. al. [30] found that the probability of detection was 14%. With five or more people shedding, the probability increased to 29% and further increased to 49% with 15 or more people shedding MPXV. Additionally, when the virus was not detected, the authors found that the probability that fewer than five people were shedding MPXV was 95%. This is in concordance with the findings of this study. Negative results, despite the high sensitivity of the assay, suggest a very low number of infected individuals, as was confirmed by the last positive reported case more than 6 months prior to the wastewater sampling campaign.

Some caution is needed when interpreting the results of mpox WWS, as it is possible to detect positive samples due to animal reservoirs of MPXV from different rodents and other small mammals such as mice and rats [32], but currently, no evidence is available that these animal reservoirs exist in non-endemic countries outside Africa [13].

Although the MPXV within the timeframe of sampling has not been detected in wastewater in Slovenia, we had an action plan in place that would be initiated based on the detection of the MPXV in the wastewater sample. Interventions would focus on increasing awareness among MSM groups and searching for positive mpox cases, taking into account the stigma associated with this disease. Awareness campaigns for MSM groups would be carried out in the catchment area of the positive sample WWTP and in public spaces where MSM people gather. As a national public health organisation, to reach the target groups, we would connect with recognised LGBTQ+ organisations and organisers of LGBTQ+ mass gatherings, while providing information on the prevention of mpox through several different communication channels that would address MSM groups, with a careful communication in order to avoid stigmatisation of this population group [33]. Besides increased preventive behaviour among MSM people, this may also lead to visiting a doctor at an earlier stage of the disease, which would help in the detection of positive mpox cases. If a positive mpox sample was detected in the wastewater, we would also urge the infectious diseases departments of the regional hospitals to be more vigilant in the management of patients for possible signs and symptoms of mpox.

Considering the good mpox epidemiological situation in Slovenia, as well as in neighbouring countries [3], we currently do not see the need for further monitoring of mpox in wastewater and the introduction of routine WWS for mpox. We recommend that the developed system be retained in the case of a recurrent epidemiological situation when it is assessed that emergency WWS of mpox would be reasonable. This refers to possible new major mpox outbreaks in European countries or to a worsening of the mpox epidemiological situation in Slovenia. The emergency WWS system for mpox in Slovenia that way could be reactivated in the event of an alert from the WHO or the European Centre for Disease Prevention and Control regarding increased public health risks of mpox spreading in Europe. In this event, we would conduct monitoring of mpox in wastewater, which has already been described in our study. The system of emergency WWS for mpox could also be reactivated in the event of a positive mpox case detected by clinical surveillance in Slovenia, for which we would in an epidemiological survey identify potential unidentified high-risk contacts or confirm secondary unlinked mpox cases. The wastewater sampling for mpox in this event would be adjusted according to the number of confirmed mpox cases, the locations of cases, and other observations from the epidemiological survey regarding high-risk contacts. The WWS of mpox in Slovenia could be carried out at 16 WWTPs from all statistical regions of Slovenia, where routine surveillance of SARS-CoV-2 and polioviruses is performed. In the event of positive results of WWS for mpox, the initial interventions would be area-specific, as was planned for the summer season of 2023, which could be further reinforced and expanded depending on the mpox epidemiological situation in the country.

Our study contributes to the development of a methodological approach to the emergency WWS of mpox, with the purpose of integrated surveillance for public health threats and the development of a public health response in the event of mpox occurrence in wastewater. In the further development of WWS for mpox, it is necessary to address the issue of shedding viral particles in the stool of mpox-infected individuals, which has already been addressed by Chen and Bibby [34], but with the use of data on viral particle shedding in the faeces of chimpanzees instead of humans, which they cite as a major limitation. The knowledge of this information would ensure a better interpretation of the results of mpox viral DNA detection in wastewater. In this way, the established system of emergency WWS for mpox would represent an important complementary system to clinical surveillance, enabling early response and the development of effective and targeted public health interventions to halt or mitigate the possible mpox spread in the population.

## 5. Conclusions

We recommend that the developed system be retained in the case of an emergency epidemiological situation. In our experiment, a laboratory method for MPXV detection was implemented for future WWS at the national level. The methodology was selected and verified based on previous experiences with SARS-CoV-2 WWS in Slovenia and according to published methods, already used for MPXV detection in such complex matrices. Besides a robust laboratory methodology, it is important to implement surveillance at the national level with a rational approach considering the epidemiological, social, and international situation.

WWS results did not reveal any positive samples for mpox, as there were no confirmed clinical cases during this period. In the event of a positive wastewater sample, targeted public health interventions would be implemented, focusing on increasing awareness among the MSM groups and searching for positive mpox cases.

## Figures and Tables

**Table 1:** Ct values for monkeypox virus and process control in the verification step.

MPXV Inoculum Dilution	Sediment				Wastewater Sample			
	MPXV		EAV		MPXV		EAV	
	Undiluted	1:10	Undiluted	1:10	Undiluted	1:10	Undiluted	1:10
1:500	29.62	31.61	34.78	35.48	20.50	22.14	27.87	27.56
1:5000	33.41	36.66	34.04	35.15	24.21	26.31	27.95	27.78
1:50,000	32.72	*	35.25	35.49	26.90	29.36	27.30	28.24
1:500,000	*	*	35.74	36.28	30.12	31.81	28.16	28.08
1:5,000,000	*	*	35.38	36.48	33.89	*	27.98	28.10

* Undetermined; MPXV—monkeypox virus; EAV—Equine Arteritis Virus, process control.

**Table 2:** The results of the PCR analysis of wastewater samples from the epidemiological surveillance of mpox in Slovenia at the wastewater treatment plant (WWTP) Ljubljana and WWTP Koper for the monitoring period from 1 June 2023 to 30 September 2023.

Sampling Date	Wastewater Treatment Plant	Flow Rate [L/Day]	MPXV PCR Result
4 June 2023	Ljubljana	61,200,000	Negative
4 June 2023	Koper	12,360,000	Negative
11 June 2023	Ljubljana	89,426,000	Negative
12 June 2023	Koper	16,970,000	Negative
18 June 2023	Ljubljana	52,360,000	Negative
19 June 2023	Koper	13,600,000	Negative
25 June 2023	Ljubljana	43,388,000	Negative
26 June 2023	Koper	11,990,000	Negative
3 July 2023	Ljubljana	44,816,000	Negative
4 July 2023	Koper	14,382,000	Negative
10 July 2023	Ljubljana	45,783,000	Negative
11 July 2023	Koper	13,410,000	Negative
16 July 2023	Ljubljana	51,493,000	Negative
17 July 2023	Koper	12,990,000	Negative
23 July 2023	Ljubljana	44,415,000	Negative
24 July 2023	Koper	18,570,000	Negative
30 July 2023	Ljubljana	57,549,000	Negative
31 July 2023	Koper	15,410,000	Negative
6 August 2023	Ljubljana	138,822,000	Negative
7 August 2023	Koper	18,900,000	Negative
16 August 2023	Ljubljana	57,397,000	Negative
16 August 2023	Koper	**	Negative
21 August 2023	Ljubljana	53,473,000	Negative
21 August 2023	Koper	**	Negative
27 August 2023	Ljubljana	45,793,000	Negative
28 August 2023	Koper	**	Negative
3 September 2023	Ljubljana	70,545,000	Negative
4 September 2023	Koper	**	Negative
10 September 2023	Ljubljana	50,844,000	Negative
11 September 2023	Koper	**	Negative
17 September 2023	Ljubljana	47,420,000	Negative
18 September 2023	Koper	**	Negative
25 September 2023	Ljubljana	92,995,000	Negative
25 September 2023	Koper	**	Negative

** Not obtainable due to technical issues at the treatment plant; MPXV—monkeypox virus.

## Data Availability

All data and materials used in this study were collected from publicly available sources and are available upon reasonable request.
